# Selective rolling circle amplification detection of site-specific DNA 5-hydroxymethylcytosine

**DOI:** 10.3389/fimmu.2026.1960016

**Published:** 2026-09-29

**Authors:** Ningning Dong, Qing Hong, Xin Sui, Jie Yan, Yaping Gao, Zehua Wang, Yao Luo, Yanru Qin

**Affiliations:** 1 Department of Oncology, The First Affiliated Hospital of Zhengzhou University, Zhengzhou, China; 2 Department of Laboratory Medicine, Sichuan Medical Laboratory Clinical Medicine Research Center, West China Hospital, Sichuan University, Chengdu, China

**Keywords:** 5-hydroxymethylcytosine, DNA methylation, epigenetic biomarker, HiFi Taq DNA Ligase, lateral flow dipstick, rolling circle amplification, site-specific detection, tumor microenvironment

## Abstract

Site-specific 5-hydroxymethylcytosine (5hmC) has been identified as a potential biomarker involving diagnosis, prognosis, and tumor immune microenvironment across multiple cancers. However, directly distinguishing trace levels of 5hmC from its highly abundant cytosine/5-methylcytosine (C/5mC) analogs remains a technical challenge. Herein, we present a 5hmC selective ligation-rolling circle amplification (5hmC-RCA) that enables the direct, high-specificity discrimination of 5hmC from C/5mC. The high specificity of 5hmC-RCA depends on the high selectivity of HiFi Taq DNA Ligase, which exhibits robust tolerance for mismatched 5hmC/A base pairs, thereby efficiently ligating the associated nicking site. While the mismatched 5mC/A or C/A pairs fail to be recognized by HiFi Taq DNA Ligase. As a proof of principle, the 5hmC-RCA assay was evaluated for its performance using Septin9 gene, which considered as key biomarkers in the prognosis and diagnosis of CRC, as the target, demonstrating a detection sensitivity of approximately 0.5%. Notably, the assay exhibited no cross-reactivity with unmodified target even at a high background concentration of 5,000 copies per reaction. By coupling HiFi Taq DNA Ligase-mediated high selective ligation with RCA, sensitive and selective quantification of site-specific 5hmC is achieved. Due to the simplicity of 5hmC-RCA, it also has potential for use in cfDNA originating from the tumor microenvironment and immune cells.

## 1 Introduction

The identification and sensitive detection of DNA epigenetic modifications are pivotal to unraveling their intricate biological functions and underlying molecular mechanisms (1). Among the diverse repertoire of epigenetic marks, 5-hydroxymethylcytosine (5hmC) has emerged as a functionally critical mediator, exerting profound regulatory control over gene expression programs that regulate cellular development, tumor microenvironment and oncogenic transformation (2–4). Notably, activated T cells within the tumor microenvironment of both responders and non-responders can be distinguished in plasma cfDNA via 5hmC-based biomarkers (5). Furthermore, 5hmC levels are markedly diminished in tumor tissues relative to adjacent normal counterparts, a reduction attributed to the hypoxic conditions prevalent in the tumor microenvironment, which suppress the enzymatic activity of the Ten-Eleven Translocation (TET) family (6). Despite these advances, critical gaps remain in our understanding of 5hmC-mediated biological functions and their precise roles in driving disease initiation and progression (7). Thus, the development of facile, sensitive, and reliable detection strategies for 5hmC is urgently needed to address these knowledge gaps-ultimately enabling the validation of 5hmC as a robust epigenetic biomarker for cancer diagnosis and prognosis.

Many high-throughput sequencing technologies, including 5hmC DNA immunoprecipitation sequencing (hMeDIP-seq), oxidative bisulfite sequencing (oxBS-seq), chemical-assisted pyridine borane sequencing (CAPS), APOBEC-coupled epigenetic sequencing (ACE-seq), TET-assisted bisulfite sequencing (TAB-seq), and nanopore sequencing, have been developed (7–13). These approaches enable the comprehensive mapping of 5hmC across the genome. Accumulating evidence indicates variations in site-specific 5hmC levels are tightly linked to critical diseases, such as HCC (14). It highlights the potential to leverage representative specific 5hmC loci as targets for disease diagnosis (15). However, accurate quantification of site-specific 5hmC remains a significant technical challenge, due to its low abundance and structural similarity to 5-methylcytosine (5mC) and unmodified cytosine (C) (16). Due to the similar structure of 5hmC, 5mC and C, they have similar thermodynamics and kinetics, results difficult discriminate through a base-pairing based strategy directly, especially for site-specific 5hmC detection. Currently, many site-specific 5hmC detection methods have been developed, including chemical oxidation (17, 18), bisulfite treatment (19), enzyme-based (20) or CRISPR-based assay (21). However, these methods rely on cumbersome, time-consuming sample treatment process by converting 5hmC structure, thus resulting in base-pairing characteristics difference compared to C and 5mC. Such pretreatment steps inevitably induce DNA loss and degradation, which compromises quantification accuracy severely given its low abundance. Despite the growing demand for such an approach, the direct discrimination of trace 5hmC from highly abundant C/5mC remains technically difficult.

Building on prior work highlighting the exceptional selectivity of HiFi-Taq DNA Ligase (22), we report a pretreatment-free strategy for the sensitive and specific discrimination of 5hmC, leveraging ligation-mediated rolling circle amplification (5hmC-RCA). As previously established (22), HiFi Taq DNA Ligase exhibits robust tolerance for mismatched 5hmC/A base pairs, enabling efficient ligation of adjacent nicking sites-whereas mismatched 5mC/A or C/A pairs fail to be recognized and ligated. RCA is a particular DNA isothermal amplification technique that has attracted increasing attention in the field of biosensing due to its operational simplicity. By coupling this ligase-based selective recognition of 5hmC with RCA, our novel 5hmC-RCA approach achieves ultrasensitive and highly specific quantification of site-specific 5hmC, eliminating the need for cumbersome sample pretreatment steps (**Scheme 1**).

**Scheme 1:**
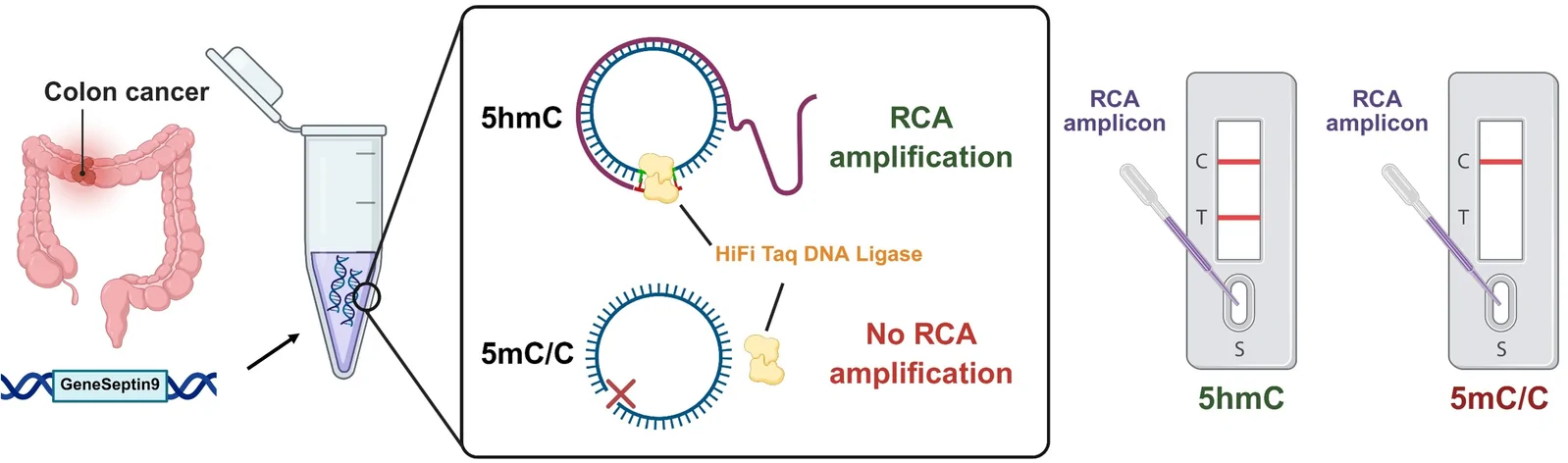
Our 5hmC-RCA approach achieves ultrasensitive and highly specific quantification of site-specific 5hmC by coupling this ligase-based selective recognition of 5hmC with RCA, which is aimed at applying to the point-of-care testing (POCT) for colon cancer.

## 2 Materials and methods

### 2.1 Materials

HiFi Taq DNA Ligase and *Bst* DNA Polymerase (Large Fragment) were purchased from New England Biolabs Ltd. All oligonucleotide sequences were synthesized by Sangon (Shanghai, China), with details listed in **Table 1**. This study was conducted in accordance with the Declaration of Helsinki and approved by the Ethics Committee of the First Affiliated Hospital of Zhengzhou University (protocol code L2025-Y252, September 26, 2025). Informed consent was obtained from all subjects involved in the study. All quantitative experiments, including clinical sample testing and optimization studies, were performed with at least three independent replicates, and the results are reported as mean ± standard deviation (SD).

**Table 1:** Oligonucleotides used for the 5hmC-RCA detection.

Name	Sequence (5′–3′)
Ligation mediator	TTGACCGCGGGGTCCTGTTGCGTCCCATGATGGCAATTGGTGCAAGTCTCGCCCATCCAGCTGCGCA
Target oligo	CATCATGTCGGACCCCGCGGTCAACGCGCAGCTGGATGGGATC
Primer	Biotin-GCCATCATGGGACGCAACA
Probe	GCGAGACTTGCACCAATT-FITC

### 2.2 Ligation by the 5hmC target

Ligation mixture was first prepared with final total volumes of 20 µl, containing 2 nM HiFi Taq DNA Ligase, 0.2 µM ligation mediator, 10 µl extracted DNA and 1x HiFi Taq DNA Ligase reaction buffer. The ligation mixture was performed with 2 cycles of 95 °C for 3 minutes for dsDNA denature and incubated at 57°C for 3 min for the denatured ssDNA hybridized to the ligation mediator and perform the ligation process.

### 2.3 RCA amplification

After ligation, 10 µl ligation products were severed as template of RCA reaction. RCA reactions contained 10 units *Bst* DNA polymerase 2.0, 1 μM 1a, 2 μM 2s, 0.5 μM templated specific probe, 50 mM Tris-HCl (pH 7.5); 10 mM MgCl_2_; 20 mM ammonium sulfate; 5% glycerol; 200 μg/mL BSA; 1 mM each dNTP. The amplification reaction mixture was incubated at 63 °C for 45 minutes, followed by 5 minutes at 95°C for inactivation. The RCA product was further demonstrated by 2% agarose gel for 1.5 h with the voltage at 100V.

### 2.4 Lateral flow detection

The LFD dipstick was obtained from Ustar Biotechnologies Co., Ltd. (Hangzhou, China). The lateral flow dipstick (LFD) comprises four functional components: a sample pad, conjugate pad, nitrocellulose membrane (25 mm × 30 cm), and absorbent pad. The sample pad and absorbent pad were treated followed the previously study (23). After pretreatment, the sample pad and conjugate pad were dried at 37°C for 4–5 hours and stored at room temperature. The four components were sequentially assembled on an adhesive backing card, with a 1.5–2 mm overlap between adjacent components to ensure efficient capillary-driven migration of the amplicon-containing solution across the dipstick.

For detection, 10 µl the amplicon solution was first applied to the sample pad, where it migrated to the conjugate pad and interacted with streptavidin-conjugated red nanoparticles. The nitrocellulose membrane was pre-functionalized with distinct antibody solutions at designated regions to form the test line (T-line) and control line (C-line). Driven by capillary action, the nanoparticle-amplicon complexes continued to migrate along the membrane, accumulating at the T-line or C-line to produce visible red bands. The presence of biotin and FITC du-labeled RCA amplicons was indicated by the appearance of a red band at the T-line position. LFD results were visually distinguishable with the naked eye within 5 minutes.

### 2.5 Statistics and reproducibility

Two-tailed Student’s t-tests were used to compare two groups. *p* < 0.05 was considered statistically significant. ns indicates no statistical significance; *p < 0.05; **p < 0.01; ***p < 0.001. Experiments were repeated at least three times.

## 3 Results

### 3.1 Principle of the 5hmC-RCA assay

HiFi Taq DNA Ligase has been shown to exhibit high selectivity for site-specific 5hmC detection (20). For the 5hmC-RCA assay, a 40-mer oligonucleotide (oligo) containing an internal “X” site (where X = C, 5mC, or 5hmC) was synthesized and served as a model target to evaluate the assay’s performance (**Figure 1A**). Additionally, a ligation mediator was designed with its 5′ end fully complementary to the target sequence, while its 3′ end was partially complementary, except for a terminal adenine (A) residue. During annealing, the 5′ and 3′ ends of the ligation mediator hybridize adjacently to the target DNA, resulting in a single-base X/A mismatch at the ligation junction.

**Figure 1:**
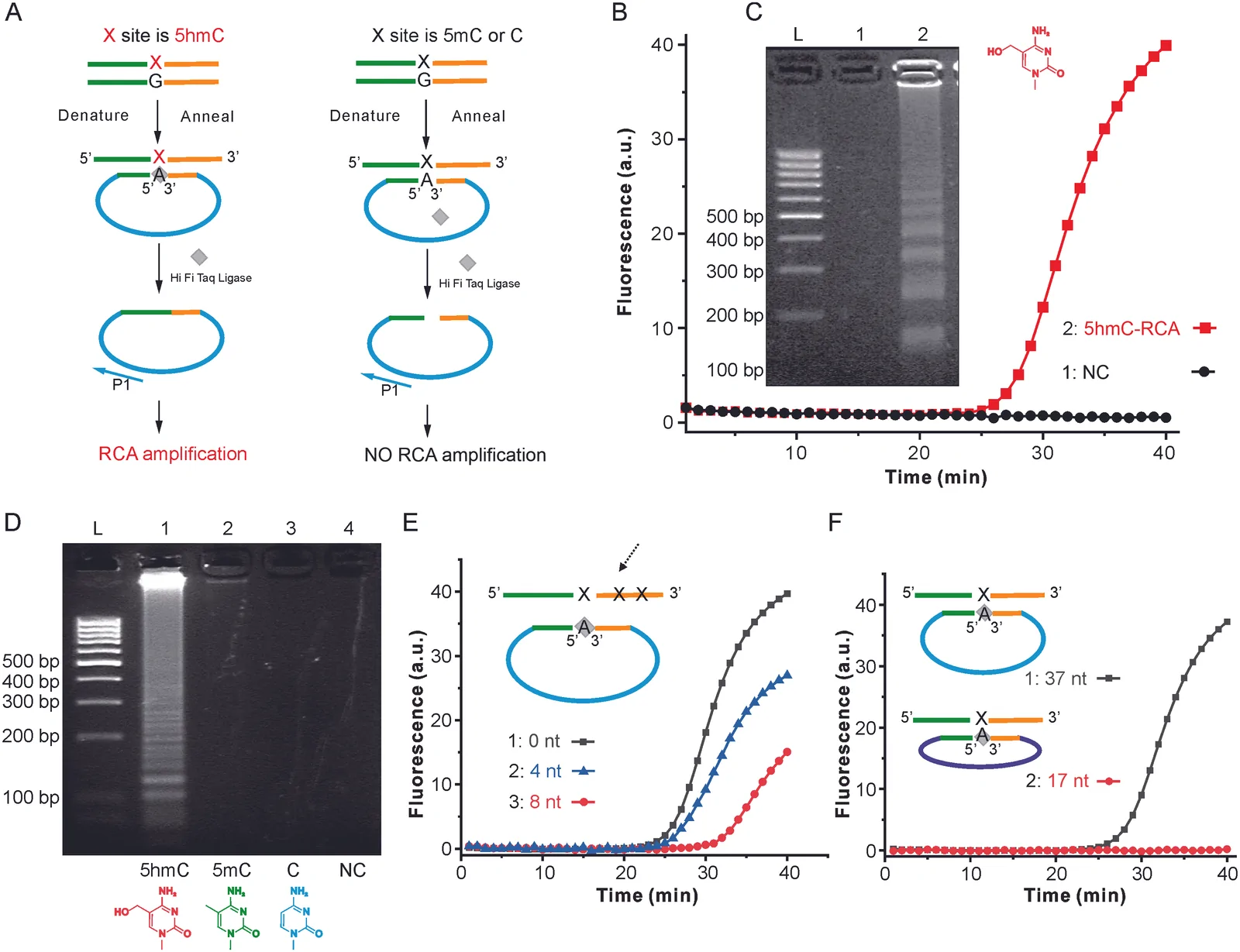
The mechanism of 5hmC-RCA in methylation detection. **(A)**. The mechanism of 5hmC-RCA; **(B)** Real-time amplification results of 5hmC-RCA. 1: 2000 copies per reaction 5hmC target (5hmC-RCA) (black squares); 2: Negative control (NC) (red circles). **(C)** Electropherograms of 5hmC-RCA (inset graph in **(B)**). L: 100 bp ladder; 1: Negative control; 2: 2000 copies per reaction 5hmC target. **(D)** Templates sharing identical sequences but harboring distinct modifications. L: 100 bp ladder; 1: 5hmC; 2: 5mC; 3: C; 4: NC. **(E)** Effect of inter-5hmC site spacing on target 5hmC detection. 1: 0 nt (black squares); 2: 4 nt (blue triples); 3: 8 nt (red circles). **(F)** Effect of ligation mediator spacer length on detection performance. 1: 37 nt (black squares); 2: 17 nt (red circles).

HiFi Taq DNA Ligase exhibits selective tolerance for 5hmC/A mismatches, but not for C/A or 5mC/A mismatches. Consequently, only site-specific 5hmC on the target DNA enables efficient ligation of the 5’ and 3’ ends of the ligation mediator, yielding a circular single-stranded DNA (ssDNA) product (**Figure 1A**). This circular ssDNA then serves as a template for subsequent rolling circle amplification (RCA). In contrast, the presence of C or 5mC at the corresponding target site prevents recognition by HiFi Taq DNA Ligase, abrogating circular ssDNA formation. Subsequently, a primer complementary to the circular ssDNA template was introduced to initiate the RCA reaction. In the presence of dNTPs and a DNA polymerase, this reaction generates abundant single-stranded DNA fragments with heterogeneous lengths (**Figures 1B, C**). These amplification products can be readily quantified using Eva Green, a fluorescent DNA-binding dye, thereby enabling the quantitative detection of site-specific 5hmC (**Figure 1B**). Thus, leveraging the selective ligation capability of HiFi Taq DNA Ligase and the high amplification efficiency of RCA, 5hmC-RCA achieves highly selective and sensitive quantification of site-specific 5hmC.

Initial specificity testing revealed that only templates bearing a 5hmC modification at the target locus yielded positive signals, while those with 5mC or C were not amplifiable, confirming the high specificity of HiFi Taq DNA Ligase (**Figure 1D**). Subsequent analysis showed that an adjacent 5hmC site located 4 nucleotides from the target site caused significant ligation inhibition (ΔC_t_ ≈ 6.5 cycles), whereas an 8-nt spacing exerted no appreciable effect (**Figure 1E**). Furthermore, a shorter spacer in the ligation mediator substantially compromised amplification (**Figure 1F**), likely due to suboptimal spatial conformation and/or reduced hybridization efficiency that impedes productive RCA template formation.

### 3.2 Optimization of formation of specific circular ssDNA

The selective recognition of 5hmC/A mismatches by HiFi Taq DNA Ligase constitutes the core mechanism of 5hmC-RCA specificity. To optimize assay performance, key parameters including ligation cycle number and ligation mediator concentration were systematically investigated. As shown in **Figure 2A**, Ct values for the 5hmC-containing target decreased with increasing ligation cycles. However, when ligation cycles exceeded 3, false-positive signals were observed with 5mC- or C-containing targets. These false positives may be attributed to decreased stringency of HiFi Taq DNA Ligase at higher cycle numbers, leading to generation of non-specific ssDNA that serves as a template for RCA amplification.

**Figure 2:**
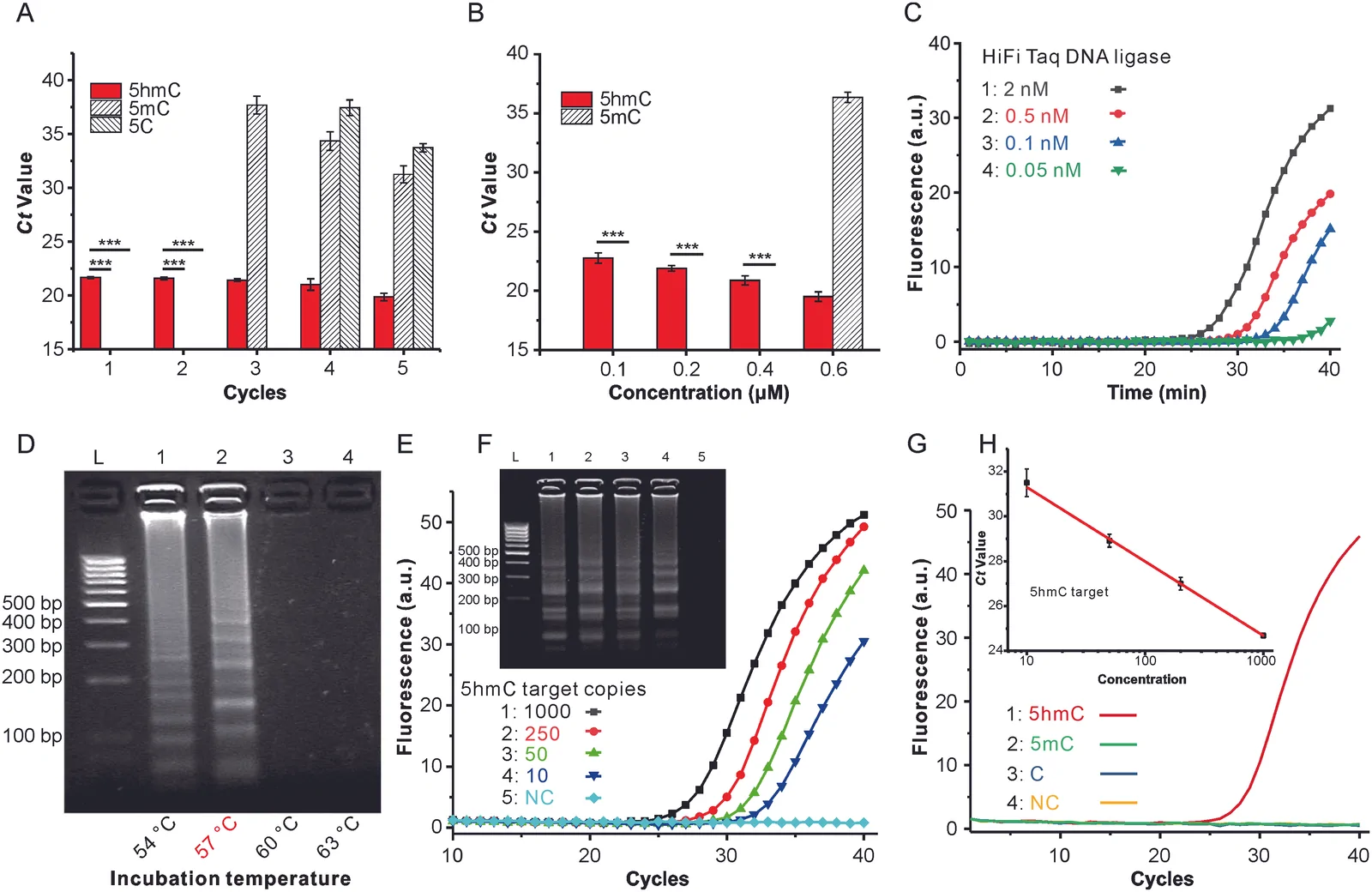
Optimization of real-time 5hmC-RCA assay. The empty grid corresponds to reactions containing the 5hmC template at a concentration of 50 copies per reaction, whereas grids marked with left-diagonal and right-diagonal lines represent reactions with the 5mC and unmodified C targets, respectively. **(A)**. Effect of HiFi Taq DNA ligase-mediated ligation cycles from 1 to 5 cycles, with the 5hmC/5mC/C target concentration at 2000 copies/reaction. All data are presented as the mean of three biological/technical replicates, and error bars denote the corresponding standard deviation. **(B)**. Effect of ligation mediator concentrations from 0.1 to 0.6 μM with the 5hmC and 5mC target concentration at 500 copies/reaction. Data represents the mean of three independent replicates, with error bars indicating the standard deviation. **(C)**. Effect of HiFi Taq DNA ligase concentration on reaction performance. 1: 2 nM (black squares); 2: 0.5 nM (red disks); 3: 0.1 nM (blue triangles); 4: 0.05 nM (green inverted triangles); **(D)** Effect of different incubation temperature of HiFi Taq DNA ligase-mediated ligation. 1: 54°C; 2: 57°C; 3: 60°C; 4: 63°C. **(E)** Real-time fluorescence curves produced by different concentrations of 5hmC target. 1: 1000 copies/reaction (black squares); 2: 250 copies/reaction (red circles); 3: 50 copies/reaction (green triangles); 4: 10 copies/reaction (blue inverted triangles); 5: ddH_2_O as a negative control (NC) (light blue lozenges). **(F)** Electropherograms of sensitivity of 5hmC-RCA [inset graph in **(E)**]. L: 100 bp ladder; 1: 1,000 copies/reaction; 2: 250 copies/reaction; 3: 50 copies/reaction; 4: 10 copies/reaction; 5: NC. **(G)** Target specificity test for 5hmC-RCA assay with the target concentration at 5000 copies/reaction. 1. 5hmC (red line); 2. 5mC (green line); 3. C (blue line); 4: NC (yellow line). **(H)** Detection of 5hmC target at varying ratios (50%, 12.5%, 2.5%, 0.5%) against a background of unmodified C target, with a total target concentration fixed at 2,000 copies per reaction [inset graph in **(G)**]. Data were the average of three replicates and error bars represent the standard deviation. The data fitted with linear regression (R^2^ = 0.99). Differences were considered statistically significant if p < 0.05. ***p < 0.001, **p < 0.01, and *p < 0.05.

The concentration of the ligation mediator was further optimized over a range of 0.2–0.6 µM, with results presented in **Figure 2B**. These data demonstrated no significant difference in assay performance when the ligation mediator concentration varied between 0.2 and 0.4 µM. However, false-positive signals were observed once the ligation mediator concentration reached 0.6 µM when using 5mC-containing targets. To determine the optimal enzyme loading, we titrated HiFi Taq DNA Ligase concentrations from 2 nM to 0.05 nM. As shown in **Figure 2C**, the detection signal decreased in a dose-dependent manner as the enzyme concentration was lowered, suggesting that ligation efficiency is directly limited by the available enzyme molecules, which in turn governs the abundance of the ligated product available for downstream amplification. We further examined the temperature dependence of the ligation reaction (**Figure 2D**). The highest detection signals were obtained within a narrow temperature range of 55–57°C, while both lower and higher temperatures led to marked signal attenuation. This temperature optimum is in good agreement with the calculated Tm of the oligonucleotide hybridization regions, indicating that proper base pairing at the ligation junction is critical for efficient enzymatic ligation.

### 3.3 Analytical performance of the 5hmC-RCA

Under the optimized experimental conditions, we evaluated the analytical performance of the 5hmC-RCA assay for site-specific 5hmC quantification by coupling with the real-time fluorescence technique. As shown in **Figures 2E**, well-defined amplification curves were generated by the 5hmC target over a range of 10, 50, 250, and 1000 copies per reaction. With increasing concentrations of the 5hmC target, the Ct values of the real-time fluorescence curves decreased gradually. The detection limit of the proposed 5hmC-RCA assay was 10 copies per reaction. The sensitivity of the 5hmC-RCA assay was further demonstrated by electropherograms, with the results shown in **Figure 2F**.

We also evaluated the specificity of the 5hmC-RCA assay toward different targets by monitoring the real-time fluorescence responses. As shown in **Figure 2G**, 5,000 copies of the 5hmC target produced a well-defined real-time fluorescence curve, whereas there was no amplification for 5mC or C targets. Therefore, the proposed 5hmC-RCA assay exhibits good selectivity for 5hmC quantification at target concentrations of up to 5,000 copies. We further assessed the selectivity of the 5hmC-RCA assay by quantifying the 5hmC target in spiked-template systems, wherein 5hmC and unmodified C targets were combined at varying ratios while the total target concentration was fixed at 2,000 copies per reaction (**Figure 2H**), the *Ct* values of independent detection were provided in **Supplementary Table 1**. These data demonstrated that the C target exerted no significant interference on 5hmC quantification; notably, as little as 0.5% 5hmC could be detected even against a high-abundance background of unmodified C.

### 3.4 LFD detection for 5hmC-RCA product

In addition to real-time fluorescence-based detection of 5hmC-RCA products, lateral flow dipstick (LFD) detection was also employed as an alternative readout strategy. For LFD-based analysis, a biotin-labeled primer complementary to the HiFi Taq DNA Ligase-generated circular ssDNA template was used to initiate RCA amplification (**Figure 3A**). Leveraging the strand displacement activity of Bst DNA polymerase 2.0 (24), extension of the biotinylated primer yielded single-stranded DNA (ssDNA) products with tandem repeat sequences. Subsequently, a 3’-FITC-labeled probe hybridized specifically to these biotinylated ssDNA amplicons, forming biotin/FITC dual-labeled complexes. Only these dual-labeled complexes yielded a positive signal on the LFD, where anti-FITC antibodies were immobilized at the test line (**Figure 3B**). The result is indicative of successful RCA amplification and the presence of site-specific 5hmC in the original sample. By contrast, when C/5mC-containing templates were used, the biotin-labeled primer could bind to the ligation mediator and extend, but the reaction terminated at the mediator’s termini. In the absence of a circular ssDNA template, the resulting linear extension products failed to hybridize to the FITC-labeled probe, precluding the formation of biotin/FITC dual-labeled complexes and thus producing no test line signal.

**Figure 3:**
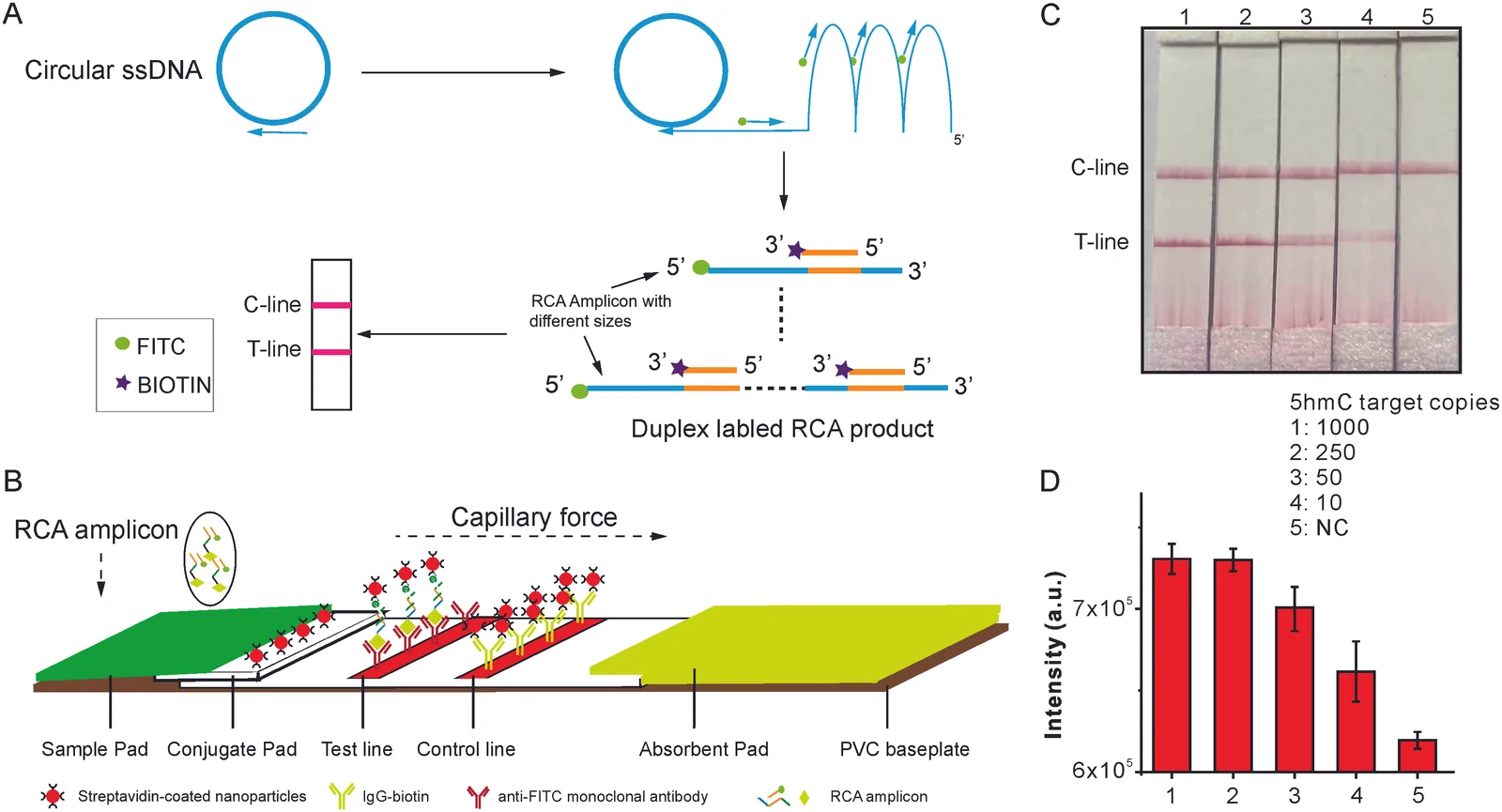
**(A)** The mechanism of 5hmC-RCA LFD detection. After HiFi Taq DNA Ligase-mediated ligation, a biotin labeled primer hybridized to the circular ssDNA leading to the generation of RCA product with tandem repeat sequences. Then a 3’end FITC labeled probe binds to the RCA product and finally detected by LFD. **(B)** The structure of lateral flow dipstick. **(C, D)** The sensitivity of 5hmC-RCA LFD results using the 5hmC target. 1: 1000 copies/reaction; 2: 250 copies/reaction; 3: 50 copies/reaction; 4: 10 copies/reaction; 5: ddH_2_O as a negative control (NC). Representative photos **(C)** and the bar graphs for the intensity of test lines **(D)** for the 5hmC-RCA-LFD assay at variable concentrations.

The sensitivity of the 5hmC-RCA assay using LFD was evaluated using a serial dilution series of 5hmC target, with concentrations ranging from 1000 to 10 copies per reaction. As shown in **Figure 3C**, the 5hmC target was detectable at a limit of detection (LOD) as low as 10 copies per reaction. The sensitivity of LFD-based detection has the same sensitivity of real-time detection. **Figure 3D** presents the results for 5hmC-RCA-LFD, which shows increasing intensity of the test line with the target concentration at the low levels. The test line intensity was seen to decrease to 50 copies/reaction, in consistence with the amplification suppression observed in **Figure 3C**.

### 3.5 Detection of site-specific 5hmC in genomic DNA

Aberrant DNA modifications, including 5hmC and 5Mc, are widely recognized as clinically validated, early molecular events that drive the initiation and progression of malignant tumors (25). To assess the potential of 5hmC as a tumor biomarker, we employed the 5hmC-RCA-LFD assay to characterize Septin9, a well-established colorectal cancer (CRC)-associated gene, and to elucidate the relationship between locus-specific 5hmC patterns and colorectal tumorigenesis (25, 26). For proof-of-principle validation, 10 formalin-fixed paraffin-embedded (FFPE) tissue samples (detailed sample information and the extracted DNA data are presented in **Supplementary Table 2** and **Supplementary Table 3**, respectively.) and previously reported 5hmC-specific loci were selected for analysis followed the workflow described in **Figure 4A**.

**Figure 4:**
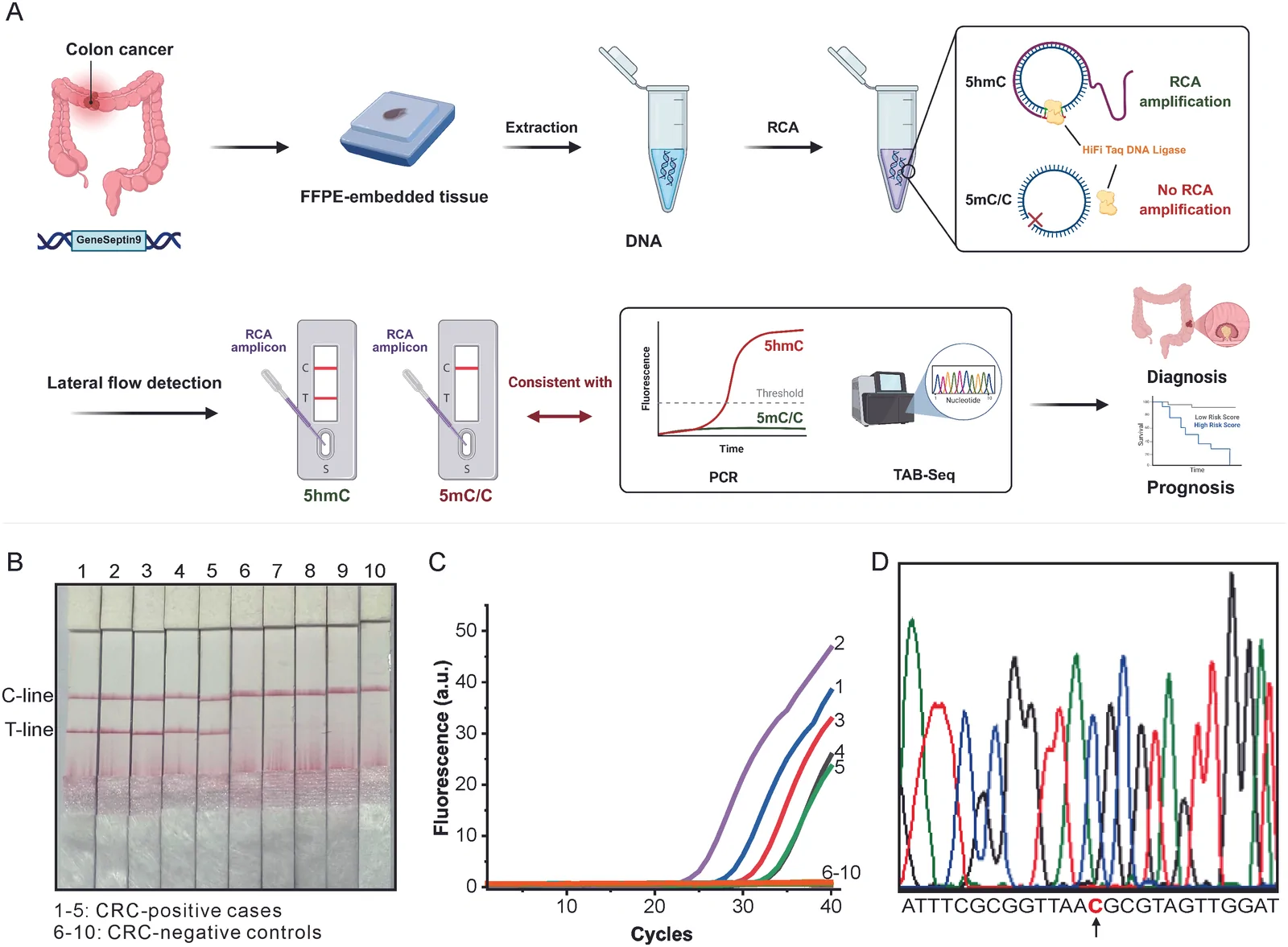
**(A)** The comparative analysis of the same 5hmC site was performed using three distinct detection methods on CRC FFPE samples. **(B)** Results of 10 clinical FFPE samples in the 5hmC-RCA LFD assay. In total, 10 samples were evaluated, with samples 1–5 representing CRC-positive cases and samples 6–10 representing CRC-negative controls. **(C)** The corresponding clinical specimens from **(B)** were subjected to standard TET-conversion-based qPCR amplification for detection. **(D)** Represent standard TET-assisted bisulfite sequencing results for 5hmC modification within Septin9 gene. The red C indicated the specific location.

First, 1 ng extracted DNA from each sample was used as a template for the 5hmC-RCA-LFD assay, with corresponding results presented in **Figure 4B**. Then 30 ng Extracted genomic was processed using a standard TET-assisted bisulfite qPCR and sequencing (TAB-Seq) protocol, followed by amplification via bisulfite-specific PCR (12), (**Figure 4C**) and subsequent validation via Sanger sequencing (**Figure 4D**). The detection results obtained by 5hmC-RCA-LFD and TET-assisted bisulfite qPCR, using pathological diagnosis as the gold standard, are summarized in **Supplementary Table 4**. Concurrently, 5hmC-RCA-LFD assay yielded identical detection outcomes to Sanger sequencing for all clinical samples tested, confirming its capability for locus-specific 5hmC detection in FFPE specimens. Nevertheless, expanded validation with larger cohorts of clinical samples will be indispensable to facilitate the assay’s future clinical translation and practical application.

## 4 Discussion

The metabolic characteristics of the tumor microenvironment exert a profound impact on 5hmC abundance. Hypoxia, a hallmark of solid tumors, imposes a direct constraint on TET enzymatic activity owing to the oxygen-dependent nature of the catalytic reaction, thereby diminishing 5hmC production (27). Concurrently, gain-of-function mutations in IDH1/2 result in the aberrant accumulation of 2-hydroxyglutarate, an oncometabolite that competitively inhibits TET enzymes and potentiates the global loss of 5hmC (28). Beyond its cell-intrinsic effects, the TET-mediated epigenetic machinery also shapes the immune landscape (29). Moreover, 5hmC signature derived from cfDNA can effectively reflect the activation status of T cells within the tumor microenvironment, offering a novel avenue for non-invasive immune monitoring (5). Consequently, the establishment of simple, highly sensitive, and reproducible approaches for 5hmC detection is critically needed to address these outstanding knowledge gaps.

Here, we have developed a robust 5hmC-RCA assay that enables the selective discrimination of 5hmC from C) and 5mC, leveraging the unique selectivity of HiFi Taq DNA Ligase. This enzyme preferentially tolerates 5hmC/A mismatches while rejecting 5mC/A or C/A mismatches at the ligation junction. By integrating this 5hmC-specific ligation step with rolling circle amplification (RCA), the assay achieves accurate quantification of site-specific 5hmC without the need for any sample pretreatment procedures. As such, the 5hmC-RCA assay features facile and streamlined operation, effectively circumventing DNA loss or degradation that typically accompanies the complex processing workflows of conventional 5hmC detection methods.

The HiFi Taq DNA Ligase exhibits an unusual but highly favorable substrate discrimination property: it readily seals a nick when the base opposite the ligation junction is 5hmC paired with an incorporated dATP, yet fails to ligate when 5mC or unmodified C occupies the same position. Unlike bisulfite sequencing (BS-seq), which cannot distinguish 5mC from 5hmC, or oxidative bisulfite sequencing (oxBS-seq) and TET-assisted bisulfite sequencing (TAB-seq), which require harsh chemical or enzymatic treatments that cause substantial DNA damage and input-material loss, our ligation-based discrimination operates under mild isothermal conditions and preserves template integrity throughout the entire workflow. This feature is particularly advantageous for precious or limited clinical specimens, where sample availability is often the primary bottleneck for downstream analyses.

Structurally, 5hmC significantly enhances intramolecular base-stacking. Even when paired with a mismatched Adenine, the presence of 5hmC maintains a remarkably rigid, stable, and un-melted local duplex structure at high temperatures. Because the nick doesn’t fray, the enzyme reads the structure as a standard, fully continuous duplex and completes the bond formation (30). However, for 5fC, the highly rigid, electron-withdrawing formyl group (-CHO) alters the electronics of the pyrimidine ring entirely (31). It changes the geometry of the base pair, induces a severe dipole moment, and causes a structural clash in the HiFi Taq DNA Ligase’s active site that completely suppresses ligation activity.

Through systematic optimization of ligation cycle number and ligation mediator concentration, our 5hmC-RCA assay attained a high-sensitivity detection limit of 10 copies per reaction for site-specific 5hmC targets, while displaying complete resistance to cross-reactivity with 5,000 copies per reaction of unmodified C or 5mC background. The assay retained the capacity to reliably identify 0.5% 5hmC fractions even in the presence of a high-abundance unmodified sequence background, underscoring its exceptional specificity for 5hmC. Furthermore, the 5hmC-RCA amplicons support rapid, instrument-free readout via LFD detection with the naked eye within 2 h, rendering the entire procedure amenable to point-of-care or resource-limited settings where conventional sequencing or chromatographic instrumentation is unavailable. A comprehensive overview of existing 5hmC detection approaches, along with a comparative evaluation of their respective performance characteristics, is summarized in **Supplementary Table 5**.

## 5 Conclusions

In summary, the 5hmC-RCA strategy described herein provides a robust tool for 5hmC analysis, featuring distinct advantages including straightforward operation, high sensitivity, and excellent specificity. Given that cfDNA-derived 5hmC signatures can reflect T-cell activation states within the TME and predict immunotherapy responses, the low-input requirement and pretreatment-free nature of 5hmC-RCA render it particularly well-suited for serial liquid biopsy monitoring in immuno-oncology settings. It should be noted, however, that the current study presents preliminary findings regarding the application of this 5hmC-RCA strategy for the selective detection of site-specific 5hmC. For real-world clinical sample applications, further optimization of reaction conditions may still be necessary to mitigate potential interference and ensure accurate quantification, particularly genomic DNA extracted from clinical tissues or liquid biopsies. Looking forward, this promising technique holds potential for the comprehensive analysis of locus-specific 5hmC when integrated with diverse devices, such as microfluidic platforms and various biosensors. The integration of 5hmC epigenetic information with microfluidic analytical platforms holds profound implications for clinical translation. First, cfDNA-based 5hmC profiling has demonstrated promising potential for non-invasively monitoring the activation status of immune cells within the tumor microenvironment and for predicting responses to immunotherapies. Second, microfluidic technologies offer robust technical support for high-throughput 5hmC detection with minimal sample requirements, thereby facilitating the transition of 5hmC from a tissue-based marker to a clinically actionable liquid biopsy biomarker. Collectively, the convergence of 5hmC biology, microfluidic engineering, and tumor microenvironment research not only deepens our understanding of tumor epigenetic heterogeneity but also charts a translational pathway from mechanistic discovery to precision diagnostics and, ultimately, to individualized therapeutic decision-making.

## Data Availability

The raw data supporting the conclusions of this article will be made available by the authors, without undue reservation.
