# Supplementary material for: Selective rolling circle amplification detection of site-specific DNA 5-hydroxymethylcytosine
Source: Front Immunol. 2026 Sep 29;17:1960016. doi: 10.3389/fimmu.2026.1960016 (PMC13658510; doi:10.3389/fimmu.2026.1960016)
Supplement: Supplementary file 1 [file DataSheet1.docx]

Supplementary Material

**Selective rolling circle amplification detection of site-specific DNA 5-hydroxymethylcytosine**

**Ningning Dong^1*^, Qing Hong^1^, Xin Sui^1^, Jie Yan^1^, Yaping Gao^1^, Zehua Wang^1^, Yao Luo^2*^ and Yanru Qin^1*^**

^1^Department of Oncology, The First Affiliated Hospital of Zhengzhou University, Zhengzhou 450000, China

^2^Department of Laboratory Medicine, Sichuan Medical Laboratory Clinical Medicine Research Center, West China Hospital, Sichuan University, Chengdu 610041, China

*** Correspondence:**

Ningning Dong

m15138753859@163.com

Yao Luo

luoyao@scu.edu.cn

Yanru Qin

yanruqin@163.com

**Supplementary Table 1. The *Ct* value of 3 independent detection.**

| **[conc]** | **1** | **2** | **3** | **AVE±SD** |
| --- | --- | --- | --- | --- |
| 50% | 24.56 | 24.74 | 24.75 | 24.68±0.11 |
| 12.5% | 27.3 | 26.74 | 26.97 | 27.00±0.28 |
| 2.5% | 29.19 | 28.93 | 28.62 | 28.91±0.29 |
| 0.5% | 30.79 | 31.85 | 31.86 | 31.50±0.61 |

**Supplementary Table 2. Clinical diagnosis results of clinical patients.**

| **No.** | **Gender** | **Age** | **5hmC-RCA-LFD** | **TET-PCR (*Ct* value)** | **Pathology** | **Test Result Consistency** |
| --- | --- | --- | --- | --- | --- | --- |
| S1 | Female | 50 | Positive | 22.34 | Positive | Yes |
| S2 | Male | 52 | Positive | 26.56 | Positive | Yes |
| S3 | Female | 54 | Positive | 28.85 | Positive | Yes |
| S4 | Male | 60 | Positive | 31.45 | Positive | Yes |
| S5 | Male | 58 | Positive | 31.25 | Positive | Yes |
| S6 | Female | 57 | Negative | No Amplification | Negative | Yes |
| S7 | Female | 59 | Negative | No Amplification | Negative | Yes |
| S8 | Female | 60 | Negative | No Amplification | Negative | Yes |
| S9 | Male | 62 | Negative | No Amplification | Negative | Yes |
| S10 | Male | 63 | Negative | No Amplification | Negative | Yes |

**Supplementary Table 3. Amount of double-stranded cell-free DNA extracted from FFPE samples.**

| **Sample number** | **concentration (Plasma, ng/ml)** |
| --- | --- |
| S1 | 25.79 ± 0.61 |
| S2 | 27.60 ± 0.66 |
| S3 | 27.87 ± 0.57 |
| S4 | 28.76 ± 0.57 |
| S5 | 29.66 ± 0.57 |
| S6 | 23. 55 ± 0.33 |
| S7 | 26.69 ± 0.44 |
| S8 | 26.69 ± 0.44 |
| S9 | 26.69 ± 0.44 |
| S10 | 26.69 ± 0.44 |

**Supplementary Table 4. The diagnostic accuracy for 5hmC-RCA-LFD and TET-PCR against Pathology.**

|  |  | Pathology | | Sensitivity | Specificity |
| --- | --- | --- | --- | --- | --- |
|  |  | POS | NEG |  |  |
| 5hmC-RCA-LFD | POS | 5 | 0 | 100% | 100% |
|  | NEG | 0 | 5 |  |  |
| TET-PCR | POS | 5 | 0 | 100% | 100% |
|  | NEG | 0 | 5 |  |  |

**Supplementary Table 5. Comparison of the existing 5hmC detection methods.**

| Method | Resolution | Advantages | Disadvantages |
| --- | --- | --- | --- |
| TET-Assisted Bisulfite Sequencing | Single-base | Directly distinguishes 5mC from 5hmC | DNA degradation; high cost; requires large DNA input |
| Oxidative Bisulfite Sequencing | Single-base | Indirectly distinguishes 5mC from 5hmC | Requires parallel BS-Seq experiment; complex and costly; DNA degradation |
| Hydroxymethylated DNA Immunoprecipitation Sequencing | Non-single-base | Simple, efficient; cost-effective;  suitable for large-scale screening | High background noise; sequence bias; antibody quality-dependent |
| Hydroxymethylation Selective Chemical Labeling | Non-single-base | Bisulfite-free; high specificity;  genome-wide coverage; low cost | Requires large DNA input |
| TET-Assisted Pyridine Borane Sequencing | Single-base | No DNA degradation; high specificity;  direct readout | TET enzyme activity-dependent; relatively lower resolution |
| APOBEC-Coupled Epigenetic Sequencing | Single-base | Bisulfite-free; high sensitivity and specificity; low input | Technically may be demanding |
| Enzymatic Methylation Sequencing | Single-base | Bisulfite-free; minimal DNA damage; more uniform GC coverage | High cost |
| Nanopore Sequencing | Single-base (theoretical) | No amplification or bisulfite conversion;  ultra-long reads | Higher error rate; high cost and high sequencing depth required |
| SMRT Sequencing | Single-base (theoretical) | Long reads; single-molecule real-time sequencing | Higher error rate; high cost |
| 5hmC-RCA | Single-base | Low DNA input requirement; | lacks genome-wide coverage |
| EDD-5hmC assay | Non-single-base | Low DNA input requirement; | lacks genome-wide coverage |
